# Machine learning provides individualized prediction of outcomes after first complete remission without allo‐HSCT consolidation in adult acute myeloid leukemia—A HARMONY study

**DOI:** 10.1002/hem3.70461

**Published:** 2026-08-30

**Authors:** Alberto Hernández‐Sánchez, Javier Martínez Elicegui, Eric Sträng, Axel Benner, Marta Sobas, Ángela Villaverde Ramiro, Amin T. Turki, María Abáigar, Gastone Castellani, Jurjen Versluis, Ian Thomas, Jesse M. Tettero, Rabea Mecklenbrauck, Joaquín Martínez‐López, Marta Pratcorona, Guillermo Sanz, Maria Teresa Voso, Sören Lehmann, Christoph Röllig, Klaus H. Metzeler, Konstanze Döhner, Michael Heuser, Torsten Haferlach, Peter J. M. Valk, Jesús María Hernández‐Rivas, Brian J. P. Huntly, Gert Ossenkoppele, Hartmut Döhner, Lars Bullinger

**Affiliations:** ^1^ Hematology Department University Hospital of Salamanca Salamanca Spain; ^2^ Institute of Biomedical Research of Salamanca (IBSAL) Salamanca Spain; ^3^ Cancer Research Center of Salamanca (USAL‐CSIC) Salamanca Spain; ^4^ Charité Universitätsmedizin Berlin Berlin Germany; ^5^ Division of Biostatistics German Cancer Research Center (DKFZ) Heidelberg Germany; ^6^ Department of Hematology, Collegium Medicum in Bydgoszcz Nicolaus Copernicus University in Toruń Bydgoszcz Poland; ^7^ HARMONY Alliance Foundation Salamanca Spain; ^8^ Marienhospital University Hospital Ruhr‐University Bochum Bochum Germany; ^9^ Institute for AI in Medicine University Hospital Essen Essen Germany; ^10^ Department of Medical and Surgical Sciences (DIMEC) University of Bologna Bologna Italy; ^11^ Erasmus MC Cancer Institute University Medical Center Rotterdam Rotterdam The Netherlands; ^12^ Centre for Trials Research Cardiff University Cardiff United Kingdom; ^13^ Department of Hematology Amsterdam UMC Location VUMC Amsterdam The Netherlands; ^14^ Department of Hematology, Hemostasis, Oncology and Stem Cell Transplantation Hannover Medical School Hannover Germany; ^15^ Weatherall Institute of Molecular Medicine University of Oxford Oxford United Kingdom; ^16^ Hospital Universitario 12 de Octubre Madrid Spain; ^17^ Hematology Hospital de la Santa Creu i Sant Pau Barcelona Spain; ^18^ CIBERONC, Instituto de Salud Carlos III Madrid Spain; ^19^ Hospital Universitario y Politécnico La Fe Valencia Spain; ^20^ University of Rome Tor Vergata Rome Italy; ^21^ Uppsala University Hospital Uppsala Sweden; ^22^ Department of Medicine Huddinge, Karolinska Institute Stockholm Sweden; ^23^ Medical Department I University Hospital TU Dresden Dresden Germany; ^24^ University of Leipzig Leipzig Germany; ^25^ Department of Internal Medicine III University Hospital of Ulm Ulm Germany; ^26^ Department of Internal Medicine IV, University Hospital Halle (Saale) Martin‐Luther‐University Halle‐Wittenberg Halle Germany; ^27^ MLL Munich Leukemia Laboratory Munich Germany; ^28^ Department of Haematology and Cambridge Stem Cell Institute University of Cambridge Cambridge United Kingdom; ^29^ Department of Hematology, Oncology, and Tumor Immunology Charité ‐ Universitätsmedizin Berlin Berlin Germany; ^30^ German Cancer Consortium (DKTK) Partner Site Berlin, and German Cancer Research Center (DKFZ) Heidelberg Germany

## Abstract

Allogeneic hematopoietic stem cell transplantation (allo‐HSCT) is a curative treatment option for a significant proportion of patients with acute myeloid leukemia (AML), and it is generally recommended when the relapse risk without allo‐HSCT outweighs the estimated non‐relapse mortality significantly. While current recommendations for allo‐HSCT are based on risk groups, there is considerable heterogeneity within these individual categories. We analyzed 2550 intensively treated AML patients aged 18–70 with cytogenetic and next‐generation sequencing data from the HARMONY Alliance database, who did not receive allo‐HSCT in first complete remission (CR1). A non‐parametric machine learning (ML) model based on Bayesian Additive Regression Trees (BART) integrated clinical variables and genomic aberrations to provide individualized outcome estimations. External validation was performed in a cohort of 714 patients enrolled in UK‐NCRI trials. The predictive performance of the HARMONY ML model, measured by the area under the time‐dependent receiver operating curve (AUC(t)), was superior to European LeukemiaNet (ELN)2022 risk classification in estimating 5‐year overall survival (0.741 vs. 0.700), 5‐year relapse‐free survival (0.752 vs. 0.705), and 5‐year cumulative incidence of relapse (0.742 vs. 0.708), which was confirmed in the external validation cohort. Notably, the model revealed substantial heterogeneity within ELN2022 risk groups, identifying a significant proportion of favorable‐risk patients with a predicted 5‐year CIR > 40%, who could potentially benefit from allo‐HSCT in CR1. The HARMONY ML model provides individualized risk prediction in intensively treated adult AML patients and supports more tailored therapeutic decisions regarding allo‐HSCT in CR1, which should be further advanced by integrating measurable residual disease in the future.

## Graphical abstract

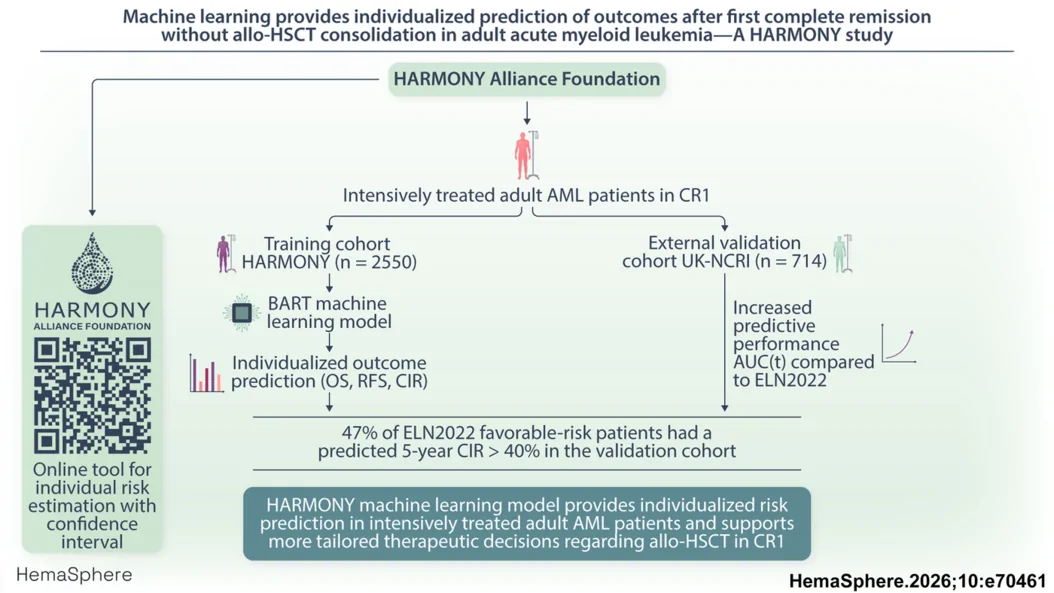

## INTRODUCTION

Acute myeloid leukemia (AML) is a heterogeneous disease in terms of underlying genomic aberrations that have a large impact on clinical outcomes.^1^ The prognostic significance of recurrent cytogenetic abnormalities has been well‐established for decades, whereas gene mutations more recently have increasingly been acknowledged for their role in predicting patient outcomes. Thus, the European LeukemiaNet (ELN) risk classification system initially considered three gene mutations in its 2010 edition.^2^ This number expanded to 6 in the 2017 edition and further increased to 13 genes in the most recent 2022 update.^3^, ^4^ This classification divides intensively treated adult AML patients into three risk categories (i.e., favorable, intermediate, and adverse) with distinct outcomes, even when allogeneic hematopoietic stem cell transplantation (allo‐HSCT) is applied.^5^ While the ELN2022 stratification system has been validated in numerous studies, further refinement can be achieved.^5^, ^6^, ^7^, ^8^ Moreover, the vast majority of AML patients present several gene mutations, and certain co‐mutational patterns have been associated with distinct outcomes, which underscores the importance of taking the whole mutational landscape into account.^1^, ^9^, ^10^

Allo‐HSCT is the pillar of treatment with curative potential for most fit adult AML patients, although a subset of patients might achieve long‐term remission without transplantation. However, allo‐HSCT entails a considerable non‐relapse mortality (NRM) due to complications related to the procedure. Therefore, a careful evaluation between the risk of disease relapse and NRM is needed for the challenging decision of whom to transplant in first complete remission (CR1). Accordingly, an AML relapse risk of >40% has been suggested for recommending an allo‐HSCT in potential candidates, provided that the predicted NRM is lower.^11^, ^12^, ^13^ Overall, ELN intermediate‐risk and adverse‐risk patients usually benefit from allo‐HSCT, while favorable‐risk patients are considered to have a cumulative incidence of relapse (CIR) of <40% and therefore allo‐HSCT is not routinely performed.^12^, ^13^ However, a subset of favorable‐risk patients might have a higher relapse risk and could also potentially benefit from allo‐HSCT, which highlights the importance of more tailored strategies.^14^

The advancements in machine learning (ML) techniques provide a novel approach to risk stratification in adult AML. Some models have demonstrated increased accuracy in predicting the probability of early death following intensive chemotherapy treatment or the chance of obtaining complete remission (CR).^15^, ^16^, ^17^ In fact, failure to achieve CR has been recognized as the most powerful predictor of overall survival (OS) in AML, highlighting the challenge of predicting outcomes for patients who have already reached CR1.^18^ However, this prediction plays a crucial role in guiding which patients should undergo allo‐HSCT in CR1.^11^, ^12^

Despite these advances, AML risk stratification remains group‐based and might not capture the relapse risk of the individual patient. This limitation influences the decision of whom to consolidate with allo‐HSCT in CR1, particularly within the ELN2022 favorable‐risk group, where a subset of patients might present a higher relapse risk that could justify transplantation, underscoring the need for individualized risk estimates to guide clinical decisions. To address this gap, we analyzed a large cohort of intensively treated adult AML patients who achieved CR1 and were not consolidated with allo‐HSCT, from the Healthcare Alliance for Resourceful Medicine Offensive against Neoplasms in Hematology (HARMONY) AML international database. We applied a non‐parametric ML approach based on Bayesian Additive Regression Trees (BART) to develop a model that provides individualized estimations of outcomes, with improved predictive performance over current risk classifications, and these findings were validated in a large external dataset that is publicly available.

## METHODS

### Training cohort

**Figure 1:**
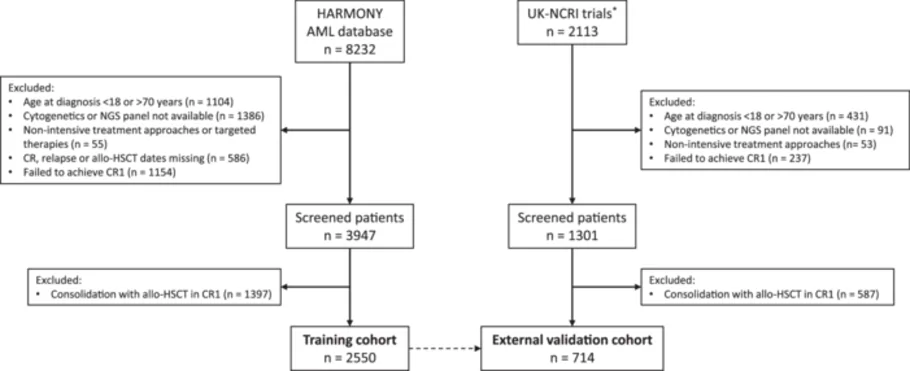
**Study flow diagram.** allo‐HSCT, allogeneic hematopoietic stem cell transplantation; AML, acute myeloid leukemia; CR, complete remission; CR1, first complete remission; NGS, next generation sequencing. *Tazi et al.^19^

The HARMONY Alliance AML database was used for this study, which included 8232 patients at the time of the analysis. Inclusion criteria were patients aged 18–70 years, who received intensive chemotherapy regimens, with an available cytogenetic study and next‐generation sequencing (NGS) myeloid panel at AML diagnosis. Exclusion criteria were missing information regarding CR, relapse or allo‐HSCT dates, administration of targeted therapies (e.g., anti‐CD33 antibodies, *FLT3*, or *IDH1/2* inhibitors), or failure to achieve CR1. Finally, patients who underwent allo‐HSCT in CR1 as consolidation treatment were also excluded (Figure 1).

A total of 2550 adult AML patients were selected for the training cohort of this analysis, contributed by eight European centers or cooperative groups. The training cohort comprised patients from prospective multicenter clinical trials of the German–Austrian AML Study Group (AMLHD98A, AML‐HD98B, and AMLSG‐07‐04) and HOVON‐SAKK (HO102, HO103, and HO132), as well as patients contributed by the Study Alliance Leukemia AML registry (Germany), the Munich Leukemia Laboratory (Germany), the AML Cooperative Group registry (Germany), the Swedish AML registry (Sweden), and the University Hospital 12 de Octubre (Spain).^1^, ^10^, ^20^, ^21^, ^22^ In addition, a multicenter cohort of patients with t(8;21)(q22;q22.1)/*RUNX1*::*RUNX1T1* AML was also included.^14^

Patient data uploaded to the HARMONY Big Data Platform underwent a thorough double‐broker pseudonymization process in compliance with the General Data Protection Regulation (GDPR). The data were then harmonized and transformed using the Observational Medical Outcomes Partnership (OMOP) Common Data Model.^23^

The study was performed in accordance with the Declaration of Helsinki and was approved by the HARMONY steering committee and the AML working group. The HARMONY research project was reviewed and authorized by the Medicinal Research Ethics Committee of the University of Salamanca (PI 2018 10 128). HARMONY has implemented a robust ethical and data‐protection framework for the secondary use of data. Prior written informed consent had been obtained from all patients at the respective HARMONY partner institutions.

### Model development and statistical analysis

BART, a non‐parametric ML model that is able to handle complex non‐linear associations and incorporate uncertainty in predictions, was used to provide individualized outcome estimations. The analysis was performed utilizing R statistical software (v3.6.3) and the BART package.^24^, ^25^, ^26^ Clinical variables (age, gender, history of prior hematological malignancy or previous cytotoxic, and/or radiation therapy) as well as cytogenetic abnormalities and gene mutations that were found in at least 10 patients in the training cohort were included in the model. CR, relapsed disease, OS, relapse‐free survival (RFS), and CIR were defined as recommended by international guidelines,^4^ although all time‐to‐event outcomes (OS, RFS, and CIR) were calculated from the date of CR1 and therefore analytical variables at diagnosis were not included in the model.

The predictive performance of the model was evaluated using 10‐fold cross‐validation, calculating the area under the time‐dependent receiver operating curve (AUC(t)) for pre‐defined time points (*t* = 1, 2, 3, 4, and 5 years) using the timeROC package,^27^ which was subsequently compared to the ELN2022 risk classification.^4^

OS and RFS were estimated using the Kaplan–Meier method, with differences in survival distributions assessed through the log‐rank test. CIR was calculated using the Aalen–Johansen method for competing risks and differences between distributions were analyzed using Gray's test. All reported P‐values were two‐sided considering the conventional 5% significance level. Further details are available in the Supplementary Appendix.

### External validation cohort

The model was externally validated using a publicly available dataset published by Tazi et al., which was not part of the HARMONY AML database at the time of the analysis.^19^ This cohort comprised adult AML patients enrolled in UK‐NCRI trials (AML17, AML16, AML11, AML12, AML14, and AML15). Of note, some of these patients received gemtuzumab ozogamicin (GO) or the *FLT3* inhibitor lestaurtinib in addition to intensive chemotherapy regimens, as part of AML17 randomizations. The inclusion and exclusion criteria for the external validation dataset matched those of the HARMONY cohort, with the previously noted exception regarding certain targeted therapies.

## RESULTS

### Patient characteristics of HARMONY cohort

The training cohort of 2550 adult AML patients in CR1 included 52% males. The median age at diagnosis was 52 years (range 18–70) and 72% were younger than 60 years (Table 1). Five percent of patients had a clinical history of prior hematological malignancy and 3% presented with therapy‐related AML. The majority of patients (53%) were classified as favorable‐risk according to the ELN2022 classification, while 25% and 22% were considered intermediate‐ and adverse‐risk, respectively. Although none of the patients received allo‐HSCT in CR1 (an exclusion criterion for this study), the 5‐year cumulative incidence of allo‐HSCT following salvage therapy was 16%. Median follow‐up was 6.4 years, with a 5‐year CIR of 52% and median OS of 5 years. Median OS after AML relapse was 7.1 months (9.6, 6.4, and 6.3 months for ELN2022 favorable‐, intermediate‐, and adverse‐risk patients, respectively; Figures S1–S3).

**Table 1:** Baseline characteristics of adult acute myeloid leukemia (AML) patients included in HARMONY database that were selected for this study (training cohort) and comparison with external validation cohort.

	Training cohort (*n* = 2550)	External validation cohort (*n* = 714)	P‐value
Female sex	1215 (47.6%)	347 (48.6%)	0.67
Median age at diagnosis in years (range)	52 [18−70]	50 [19−70]	0.002
	[Q1: 41, Q3: 61]	[Q1: 40, Q3: 59]	
Age ≥60 years	714 (28%)	143 (20%)	<0.001
AML type			0.7
De novo AML	2353 (92.3%)	662 (93.7%)	
Secondary AML	197 (7.7%)	52 (7.3%)	
Prior HM	122 (4.8%)	30 (4.2%)	
Therapy‐related AML	75 (2.9%)	22 (3.1%)	
Hemoglobin (g/dL) (*n* = 2355)	9	9.2	0.61
	[Q1 = 7.8|Q3 = 10.5]	[Q1 = 7.8|Q3 = 10.5]	
WBC (×10^9^/L) (*n* = 2524)	14.3	15	1
	[Q1 = 4.5|Q3 = 43.4]	[Q1 = 4.4|Q3 = 42]	
WBC > 100 ×10^9^/L	224 (8.9%)	63 (8.8%)	1
Platelets (×10^9^/L) (*n* = 2435)	53	57.5	0.15
	[Q1 = 29|Q3 = 98.5]	[Q1 = 31.2|Q3 = 101.8]	
Bone marrow % of blasts (*n* = 2392)	63.8%	61.5	0.45
	[Q1 = 40|Q3 = 81.5]	[Q1 = 37|Q3 = 81]	
ELN2022			<0.001
Favorable	1357 (53.2%)	319 (44.7%)	<0.001
*NPM1*‐mut without *FLT3*‐ITD	611 (24%)	170 (23.8%)	0.96
t(8;21)(q22;q22.1)/*RUNX1*::*RUNX1T1*	365 (14.3%)	66 (9.2%)	<0.001
inv(16)(p13.1q22) or t(16;16)(p13.1;q22)/*CBFB*::*MYH11*	202 (7.9%)	58 (8.1%)	0.87
bZIP in‐frame mutated *CEBPA* a	181 (7.1%)	27 (3.8%)	<0.001
Intermediate	626 (24.5%)	199 (27.9%)	0.072
Adverse	567 (22.2%)	196 (27.5%)	0.004
Most frequently mutated genes			
*NPM1*	921 (36.1%)	269 (37.7%)	0.45
*DNMT3A*	671 (26.3%)	204 (28.6%)	0.23
*FLT3*‐ITD	484 (19%)	133 (18.6%)	0.87
*NRAS*	502 (19.7%)	121 (16.9%)	0.1
*TET2*	345 (13.5%)	82 (11.5%)	0.17
5‐Year cumulative incidence of salvage allo‐HSCT (not in CR1)	16% [14%−17%]	26% [23%–29%]	<0.001
5‐Year cumulative incidence of relapse (%)	52% [50%−54%]	54% [50%−58%]	0.71
Median overall survival in years (95% CI)	4.97 [4.14−6.37]	8.38 [4.76–NA]	0.07
Median overall follow‐up in years (95% CI)	6.38 [6.14−6.58]	6.45 [6.25−6.66]	0.02

Abbreviations: allo‐HSCT, allogeneic hematopoietic stem cell transplantation; CI, confidence interval; CR1, first complete remission; ELN, European LeukemiaNet; HM, hematological malignancy; ITD, internal tandem duplication; WBC, white blood count.

^a^ CEBPA bZIP mutation status was imputed in 17.6% of patients (see Supplementary Appendix).

### Genomic landscape of HARMONY cohort

The most frequent cytogenetic abnormalities were t(8;21)(q22;q22.1)/*RUNX1*::*RUNX1T1* (14.3%), inv(16)(p13.1q22)/t(16;16)(p13.1;q22)/*CBFB*::*MYH11* (7.9%), and loss of the Y chromosome (6.9%), while 52% of patients had a normal karyotype. The most frequently mutated genes were *NPM1* (36.1%), *FLT3* in 29.9% (ITD 19%, TKD 10.9%), *TET2* (13.5%), *IDH2* (11.8%), *KIT* (8.4%), *PTPN11* (8.4%), and *RUNX1* (8%). Overall, a total of 76 genomic aberrations were present in at least 10 patients and were therefore included in the ML model (Table S1).

### HARMONY ML model

**Figure 2:**
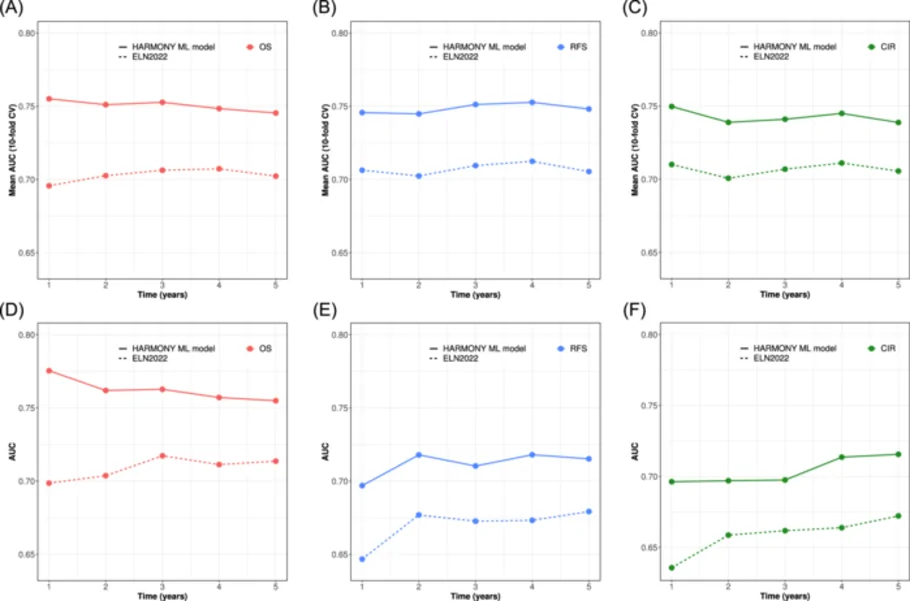
**Predictive performance of HARMONY machine learning (ML) model compared to European LeukemiaNet (ELN)2022 risk classification, measured by area under the time‐dependent receiver operating curve (AUC(t)) at 1, 2, 3, 4, and 5 years. (A)** Overall survival in training cohort. **(B)** Relapse‐free survival in training cohort. **(C)** Cumulative incidence of relapse in training cohort. **(D)** Overall survival in external validation cohort. **(E)** Relapse‐free survival in external validation cohort. **(F)** Cumulative incidence of relapse in external validation cohort. CIR, cumulative incidence of relapse; OS, overall survival; RFS, relapse‐free survival.

A BART ML model was developed in order to estimate individualized prediction of outcomes, incorporating clinical variables and the aforementioned genomic features. The performance of the HARMONY ML model for OS discrimination, as measured by mean AUC(t) after 10‐fold cross‐validation, was 0.758, 0.752, and 0.741 for 1‐, 3‐, and 5‐year OS, respectively. Conversely, ELN2022 risk classification resulted in AUC(t) values of 0.694, 0.704, and 0.700 for OS at the same time points. The predictive performance of the HARMONY ML model was also higher than ELN2022 for 1‐year RFS (AUC 0.748 vs. 0.706), 3‐year RFS (AUC 0.755 vs. 0.709), 5‐year RFS (AUC 0.752 vs. 0.705), 1‐year CIR (AUC 0.754 vs. 0.714), 3‐year CIR (0.746 vs. 0.709), and 5‐year CIR (0.742 vs. 0.708) (Figure 2, Table S2).

**Figure 3:**
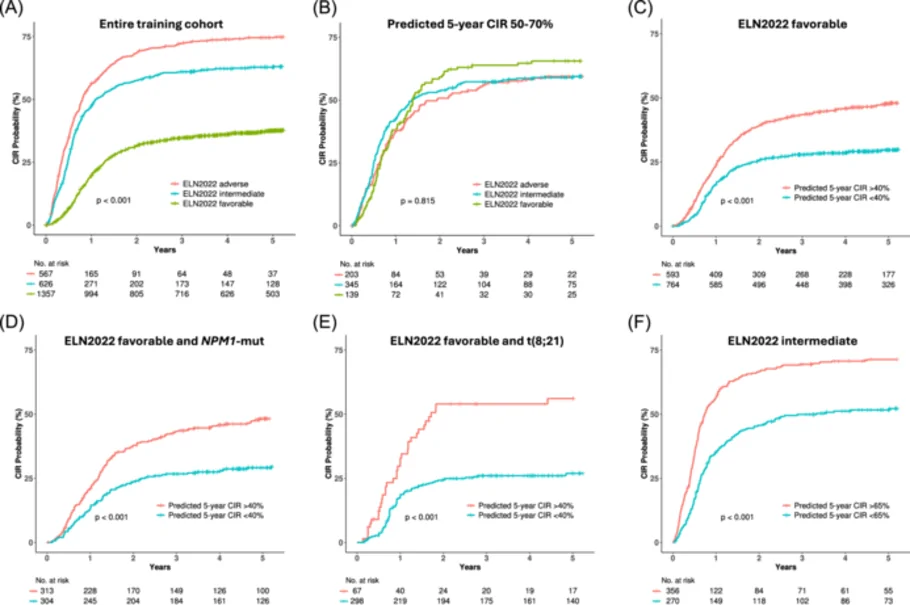
**Cumulative incidence of relapse in training cohort. (A)** Entire training cohort, stratified by European LeukemiaNet (ELN)2022 risk classification. **(B)** Only patients with predicted 5‐year cumulative incidence of relapse (CIR) of 50%–70% according to HARMONY machine learning (ML) model, stratified by ELN2022 risk classification. **(C)** ELN2022 favorable‐risk patients, stratified by predicted 5‐year CIR according to HARMONY ML model. **(D)** ELN2022 favorable‐risk patients with *NPM1* mutation, stratified by predicted 5‐year CIR according to HARMONY ML model. **(E)** ELN2022 favorable‐risk patients with t(8;21)(q22;q22.1)/*RUNX1*::*RUNX1T1*, stratified by predicted 5‐year CIR according to HARMONY ML model. **(F)** ELN2022 intermediate‐risk patients, stratified by predicted 5‐year CIR according to HARMONY ML model.

The ELN2022 classification divided the cohort into three subgroups with distinct overall cumulative incidences of relapse: 5‐year CIR was 38% (CI 35%–40%) for favorable‐risk, 63% (CI 59%–67%) for intermediate‐risk, and 75% (CI 71%–79%) for adverse‐risk patients (Figure 3A). Similarly, the HARMONY ML model predicted an overall median 5‐year CIR of 38% for ELN2022 favorable‐risk patients, with 5‐year CIR of 66% and 74% for intermediate and adverse‐risk patients, respectively. However, the ELN subgroups showed significant heterogeneity in predicted 5‐year CIR probability; the ELN2022 favorable subgroup (95% CI) included patients with an individual 5‐year CIR estimation by HARMONY ML model that ranged from 18% to 60%, the ELN2022 intermediate group patients with predicted 5‐year CIR from 46% to 86%, and the ELN2022 adverse‐risk group patients that ranged from 52% to 91% (Figure S4). Therefore, 139/1357 (10%) favorable‐risk patients, 345/626 (55%) intermediate‐risk patients, and 203/567 (36%) adverse‐risk patients according to ELN2022 classification had a predicted 5‐year CIR between 50% and 70% according to the HARMONY ML model. Remarkably, the actual CIR probability of these subgroups, as estimated by the Aalen–Johansen method, was similar: 5‐year CIR of 66%, 59%, and 59% for ELN2022 favorable‐, intermediate‐, and adverse‐risk, respectively (P = 0.815) (Figure 3B).

The overall averaged 5‐year CIR probability of the ELN2022 favorable‐risk subset was 38%. However, the HARMONY model predicted an individual 5‐year CIR > 40% in 593 of these patients. Accordingly, the 5‐year CIR probability of this subgroup, estimated by the Aalen–Johansen method, was significantly higher than the remainder of the ELN2022 favorable‐risk with a predicted 5‐year CIR < 40% (48% vs. 30%, respectively, P < 0.001) (Figure 3C). Further dissecting this cohort, in the ELN2022 favorable subgroup with *NPM1* mutation (i.e., absence of *FLT3*‐ITD), the HARMONY model predicted an individual 5‐year CIR probability >40% in 50.9% of the patients, with a significantly higher CIR when compared to the remaining subset with predicted CIR < 40% (48% vs. 29%, P < 0.001) (Figure 3D). Of note, the *NPM1*‐mutated cohort with a predicted 5‐year CIR > 40% as calculated by the HARMONY ML model was enriched in *DNMT3A*‐mutant cases (85% vs. 9%, P < 0.001) and a clinical history of prior hematological malignancy (6.7% vs. 0.3%, P < 0.001). Conversely, a *STAG2*‐mutation was less frequent (0.3% vs. 5.6%, P < 0.001) in this poorer risk subgroup than in the subset with predicted 5‐year CIR < 40%. Similarly, for the cases with a t(8;21)(q22;q22.1)/*RUNX1*::*RUNX1T1* rearrangement, the HARMONY ML model predicted an individual 5‐year CIR > 40% in 18.4% patients, with that subgroup having a significantly higher CIR when compared to the remaining patients with a predicted CIR < 40% (56% vs. 27%, P < 0.001) (Figure 3E). The poorer risk subgroup had a higher proportion of *RUNX1*‐ (9% vs. 1.3%, P = 0.004), *DNMT3A*‐ (9% vs. 1%, P = 0.002), and *TP53*‐mutations (4.5% vs. 0%, P = 0.006). Finally, heterogeneity was also observed within the ELN2022 intermediate subgroup, as patients with predicted 5‐year CIR > 65% by the HARMONY model presented a significantly higher CIR than those with individual prediction <65% (5‐year CIR 71% vs. 52%, respectively, P < 0.001) (Figure 3F).

### External validation of HARMONY ML model

The external validation cohort of 714 adult AML patients in CR1, who had not received allo‐HSCT as consolidation treatment, demonstrated some significant differences compared to the training cohort, as patients were younger (median 50 vs. 52 years, P = 0.002; age ≥ 60 years 20% vs. 28%, P < 0.001), with a lower proportion of patients classified as ELN2022 favorable‐risk (44.7% vs. 53.2%, P < 0.001), higher percentage of adverse‐risk patients (27.5% vs. 22.2%, P = 0.004), and increased allo‐HSCT rates as salvage treatment (25.5% vs. 15.5%, P < 0.001) (Table 1). The distribution of genomic aberrations included in the HARMONY ML model in the validation cohort is indicated in Table S3.

However, despite these differences, the predictive performance of the HARMONY ML model, as measured by AUC(t), was also higher than ELN2022 in the external validation cohort across a wide‐range of metrics: 1‐year OS (0.777 vs. 0.699), 3‐year OS (0.764 vs. 0.717), 5‐year OS (0.755 vs. 0.714), 1‐year RFS (0.698 vs. 0.647), 3‐year RFS (0.713 vs. 0.673), 5‐year RFS (0.717 vs. 0.679), 1‐year CIR (0.699 vs. 0.636), 3‐year CIR (0.700 vs. 0.662), and 5‐year CIR (0.718 vs. 0.672) (Figure 2, Table S4). Model calibration, assessed in both the training and the external validation cohorts, showed close agreement between predicted and observed outcomes (Figure S5). Moreover, this superior discrimination over ELN2022 was retained when the model was restricted to genetic variables (i.e., excluding age, sex, and AML type), indicating that the improvement was mainly driven by the model's handling of genomic information rather than by the inclusion of additional clinical covariates (Figure S6).

**Figure 4:**
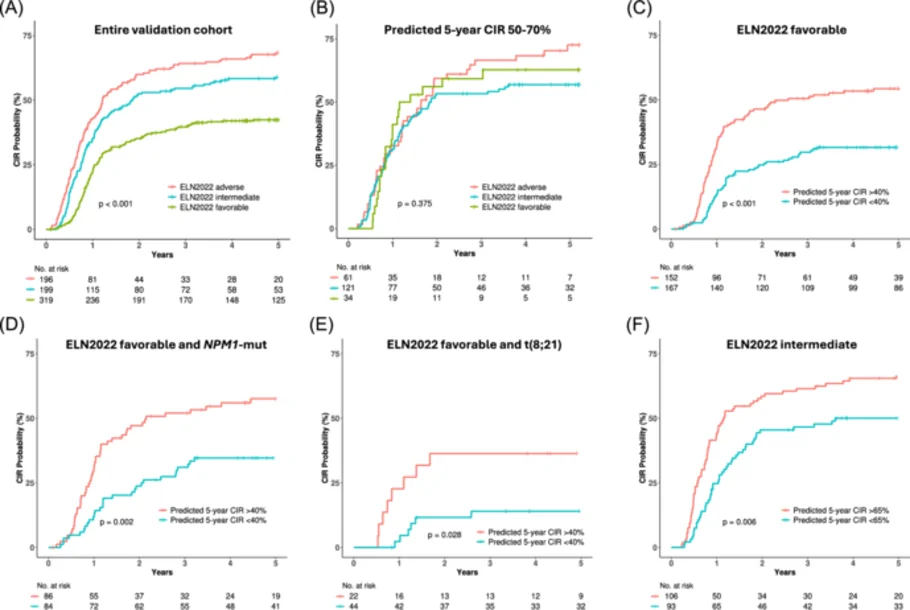
**Cumulative incidence of relapse in external validation cohort. (A)** Entire external validation cohort, stratified by European LeukemiaNet (ELN)2022 risk classification. **(B)** Only patients with predicted 5‐year cumulative incidence of relapse (CIR) of 50%–70% according to HARMONY machine learning (ML) model, stratified by ELN2022 risk classification. **(C)** ELN2022 favorable‐risk patients, stratified by predicted 5‐year CIR according to HARMONY ML model. **(D)** ELN2022 favorable‐risk patients with *NPM1* mutation, stratified by predicted 5‐year CIR according to HARMONY ML model. **(E)** ELN2022 favorable‐risk patients with t(8;21)(q22;q22.1)/*RUNX1*::*RUNX1T1*, stratified by predicted 5‐year CIR according to HARMONY ML model. **(F)** ELN2022 intermediate‐risk patients, stratified by predicted 5‐year CIR according to HARMONY ML model.

The ELN2022 classification also divided the validation cohort into three subgroups with distinct cumulative incidences of relapse: 5‐year CIR was 42% (CI 37%–48%) for favorable‐risk, 59% (CI 52%–66%) for intermediate‐risk, and 68% (CI 62%–75%) for adverse‐risk patients (Figure 4A). Individual 5‐year CIR, as predicted by the HARMONY ML model, further showed substantial heterogeneity within each ELN2022 risk category: the 5‐year CIR probability ranged (95% CI) from 20% to 56% for favorable‐risk patients, from 49% to 82% for ELN2022 intermediate‐risk patients, and was 49% to 91% in ELN2022 adverse‐risk patients (Figure S7). Accordingly, 10.6% of favorable‐risk, 60.8% of intermediate‐risk, and 31.1% of adverse‐risk patients in the validation cohort had a predicted 5‐year CIR between 50% and 70% according to the HARMONY ML model, and similarly to the test‐cohort, the actual CIR probability of these subgroups, as estimated by the Aalen–Johansen method, was similar (P = 0.375) (Figure 4B).

The overall averaged 5‐year CIR of the ELN2022 favorable‐risk subgroup was 42%, although the HARMONY ML model predicted an individual 5‐year CIR > 40% in 47.6% of patients. Remarkably, the 5‐year CIR probability of this subset, as estimated by the Aalen–Johansen method, was higher than the remainder of the cohort with predicted 5‐year CIR < 40% (54% vs. 32% for ELN2022 favorable‐risk, P < 0.001) (Figure 4C). The HARMONY ML model was able to identify that 50.6% of ELN2022 favorable‐risk patients with *NPM1*‐mut and 33.3% with t(8;21)(q22;q22.1)/*RUNX1*::*RUNX1T1* rearrangements had an individual 5‐year CIR > 40% (Figure 4D,E). Heterogeneity was also confirmed within the ELN2022 intermediate subgroup, as patients with predicted 5‐year CIR > 65% by the HARMONY ML model presented a significantly higher CIR than those with individual prediction < 65% (5‐year CIR 67% vs. 50%, respectively, P = 0.006) (Figure 4F).

### Online tool for individual risk estimation

**Figure 5:**
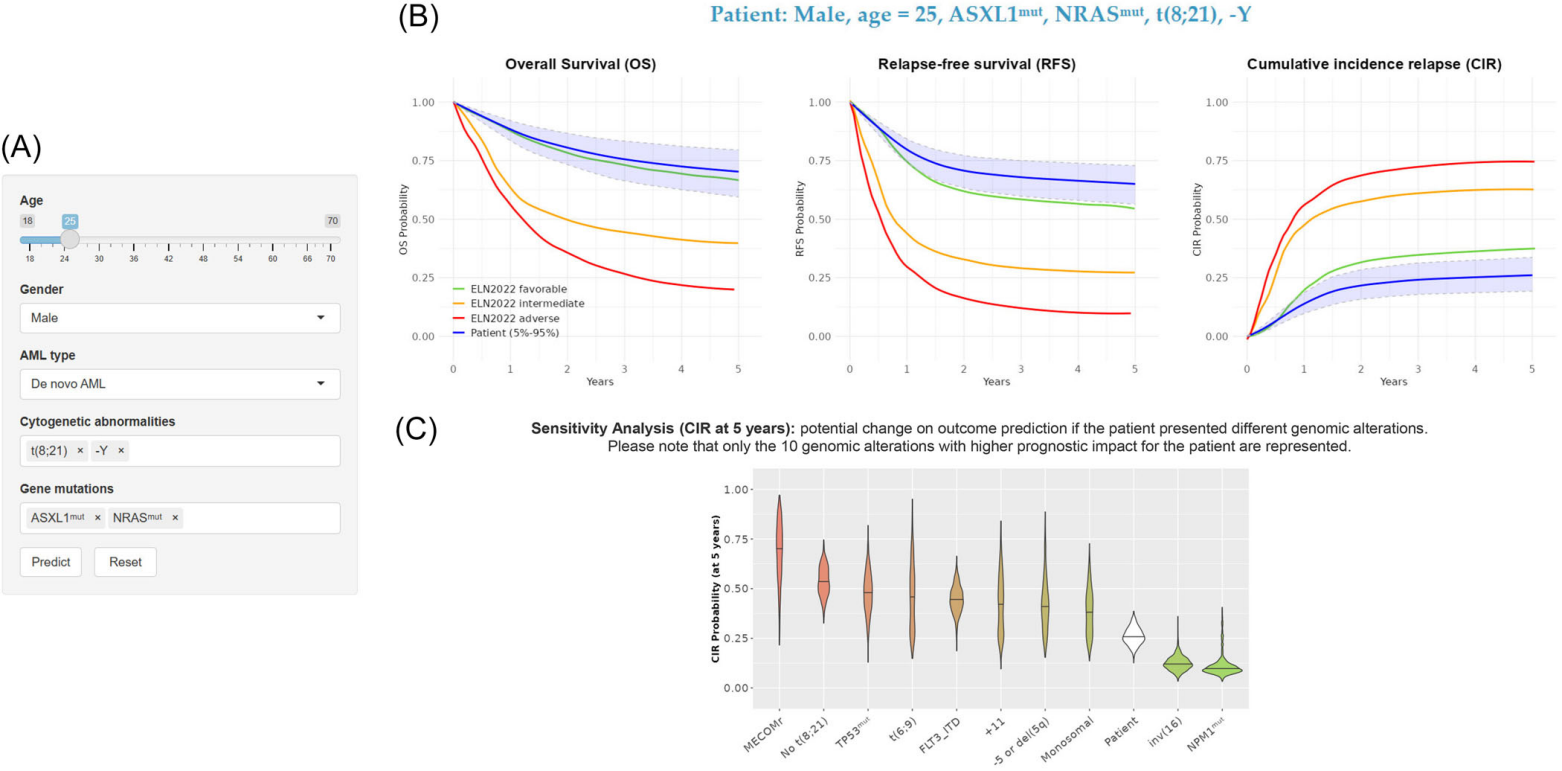
**Web‐based interface integrating HARMONY machine learning (ML) model. (A)** Data input. **(B)** Data output, including individualized patient outcome estimation (blue line), reference of European LeukemiaNet (ELN)2022 favorable‐risk patients (green line), ELN2022 intermediate‐risk patients (yellow line), and ELN2022 adverse‐risk patients (red line). All outcome estimations are calculated from the complete remission (CR) date. **(C)** Sensitivity analysis of 5‐year cumulative incidence of relapse (CIR). AML, acute myeloid leukemia; OS, overall survival; RFS, relapse‐free survival.

A web‐based interface was developed to calculate individualized estimation of outcomes according to the HARMONY ML model (https://geneticsoncohematology.com/AML_Outcome_Predictor/). Using five input variables (age, gender, AML type, cytogenetic abnormalities, and gene mutations), a 1000‐fold patient simulation is performed to provide estimation of each outcome measure (OS, RFS, and CIR) with confidence interval for each prediction (Figure 5). The interface also includes a 5‐year CIR sensitivity analysis, reflecting potential change in outcome prediction if the patient had different genomic alterations.

## DISCUSSION

Allo‐HSCT plays a crucial role as part of the curative treatment for the majority of eligible adult AML patients. Whether to proceed with an allogeneic transplant in CR1 depends on multiple factors, including disease biology, as it is generally recommended for patients with intermediate or adverse‐risk features. However, it has been suggested that a predicted relapse risk > 40% with chemotherapy alone should also be considered as an indication for allo‐HSCT.^11^, ^12^, ^13^ In our study, we analyzed the outcomes of a large dataset of potential allo‐HSCT candidates (i.e., 18–70 years, who achieved CR1 following intensive chemotherapy regimens), confirming distinct relapse risks for ELN2022 favorable‐, intermediate‐, and adverse‐risk groups. While the relapse risk without allo‐HSCT was approximately 40% in the ELN2022 favorable‐risk group, we demonstrated considerable heterogeneity within this subset of patients, with a significant proportion of favorable‐risk patients presenting a 5‐year CIR > 40% in both training and external validation datasets. This observation is relevant from the clinical perspective, as those patients could have potentially benefited from allo‐HSCT as consolidation treatment.

The HARMONY ML model was developed combining data from randomized clinical trials as well as national registries and was validated with data from the UK‐NCRI clinical trials.^19^ The predictive performance of the HARMONY ML model, measured by AUC, was superior to ELN2022 risk classification in all outcome measures (i.e., OS, RFS, and CIR) and timepoints (1–5 years), in both the training cohort and the external independent validation dataset. While previous studies have also reported improved performance compared to the ELN classification, some models rely on analyses that are not routinely performed in clinical practice, such as gene expression profiles or epigenetic features.^28^, ^29^ In contrast, the HARMONY ML model was developed using genetic tests that are recommended at AML diagnosis in current guidelines (i.e., cytogenetics and NGS myeloid panel diagnostics) and are widespread in clinical practice, facilitating its implementation to assist routine clinical decision‐making.^4^ In this regard, estimation of outcome is calculated individually, with a confidence interval provided for each prediction, and the process can be performed rapidly and in real‐time.

The decision of whom to perform allo‐HSCT in CR1 is one of the most challenging in the clinical management of adult AML, as both disease relapse risk and NRM related to the procedure need to be taken into account. While the former could be estimated with the HARMONY ML model, the latter depends on diverse factors such as patient comorbidities, donor features and management of early complications.^30^, ^31^, ^32^ Advances in donor selection, conditioning regimens, and post‐transplant care have led to a significant reduction of transplant‐related mortality over the last decades, with a 1‐year NRM of 24.4% in the 1990s decreasing to 9.5% in the 2013–2016 period.^33^ In this context, an increasing number of patients could potentially benefit from allo‐HSCT in CR1, further highlighting the importance of an evolving assessment of allo‐HSCT risk/benefit balance.

However, some aspects of our study need to be taken into account. Although it includes multiple patient cohorts from various European institutions and has been validated by an independent UK cohort, it remains a retrospective analysis that warrants prospective evaluation. The inclusion and exclusion criteria of the study contributed to a relatively homogeneous cohort of intensively treated adult AML patients who achieved CR1 and were not consolidated with allo‐HSCT, where genomic characteristics have been described as the major determinants of patient outcomes.^34^

AML treatment has evolved over the last decade, with an increasing number of targeted therapies becoming available, and increasingly deployed in the frontline setting.^35^ In our training cohort, only patients treated with intensive chemotherapy regimens were included. However, the model was validated using an external cohort, which included a proportion of patients who were also treated with GO or *FLT3* inhibitors. The HARMONY ML algorithm will require further refinement as optimal treatment evolves, as the inclusion of patients treated with additional targeted therapies or non‐intensive approaches could enhance its ability to provide even more accurate risk estimations.

In addition, our study did not incorporate assessment of measurable residual disease (MRD), which has been established as a significant prognostic factor in AML,^36^, ^37^ and could add valuable information with regard to the relapse risk of an individual patient. While novel treatment strategies have reduced the MRD positivity rate to less than 20% in certain subtypes, such as *NPM1*‐mut AML,^38^ a 3‐year CIR of up to 40% has been observed even in some MRD‐negative genomic subgroups. This suggests that the combination of genomic features at diagnosis and evaluation of treatment response by MRD measurement can provide an even more precise estimation of patient outcomes. While a dedicated MRD study is ongoing within the HARMONY consortium in a different cohort, our model can already provide valuable insights for clinically relevant decisions.

In summary, the HARMONY ML model provides individualized outcome estimation for intensively treated adult AML patients who achieve CR1 and are consolidated without allo‐HSCT. Clinicians could balance the estimated relapse risk calculated by the model against NRM evaluated by specific scores, in order to assess the relative benefit of allo‐HSCT for their patients. Moreover, to facilitate its clinical utility, the HARMONY ML model can be accessed as an online tool (https://geneticsoncohematology.com/AML_Outcome_Predictor/).

## AUTHOR CONTRIBUTIONS

**Alberto Hernández‐Sánchez**: Conceptualization; data curation; formal analysis; writing—original draft; writing—review and editing. **Javier Martínez Elicegui**: Data curation; formal analysis; methodology; visualization; writing—review and editing. **Eric Sträng**: Data curation; formal analysis; methodology; writing—review and editing. **Axel Benner**: Formal analysis; methodology; writing—review and editing. **Marta Sobas**: Data curation; writing—review and editing. **Ángela Villaverde Ramiro**: Data curation; formal analysis; methodology; visualization; writing—review and editing. **Amin T. Turki**: Data curation; writing—review and editing. **María Abáigar**: Data curation; writing—review and editing. **Gastone Castellani**: Formal analysis; writing—review and editing. **Jurjen Versluis**: Data curation; writing—review and editing. **Ian Thomas**: Data curation; writing—review and editing. **Jesse M. Tettero**: Formal analysis; writing—review and editing. **Rabea Mecklenbrauck**: Formal analysis; writing—review and editing. **Joaquín Martínez‐López**: Data curation; writing—review and editing. **Marta Pratcorona**: Data curation; writing—review and editing. **Guillermo Sanz**: Formal analysis; writing—review and editing. **Maria Teresa Voso**: Formal analysis; writing—review and editing. **Sören Lehmann**: Data curation; writing—review and editing. **Christoph Röllig**: Data curation; writing—review and editing. **Klaus H. Metzeler**: Data curation; writing—review and editing. **Konstanze Döhner**: Data curation; formal analysis; writing—review and editing. **Michael Heuser**: Data curation; writing—review and editing. **Torsten Haferlach**: Data curation; writing—review and editing. **Peter J. M. Valk**: Data curation; writing—review and editing. **Jesús María Hernández‐Rivas**: Conceptualization; data curation; formal analysis; writing—review and editing; funding acquisition. **Brian J. P. Huntly**: Conceptualization; formal analysis; supervision; writing—review and editing. **Gert Ossenkoppele**: Conceptualization; formal analysis; supervision; writing—review and editing. **Hartmut Döhner**: Conceptualization; data curation; formal analysis; supervision; writing—review and editing. **Lars Bullinger**: Conceptualization; formal analysis; writing—original draft; supervision; writing—review and editing.

## CONFLICT OF INTEREST STATEMENT

**M.S.**: honoraria from Novartis, Celgene, AOP Orphan, and AbbVie. **K.H.M.**: honoraria from AbbVie, Bristol Myers Squibb, Celgene, Janssen, Novartis, Pfizer, and Otsuka; research funding from AbbVie. **A.T.T.**: consultancy for CSL Behring, Maat Pharma, Biomarin, and Onkowissen; travel reimbursements from Neovii Biotech and Novartis. **M.P.**: honoraria from Novartis. **G.S.**: honoraria from Takeda and has participated in Ad Boards from Novartis, Celgene, AbbVie, Helsinn, and Takeda. **C.R.**: honoraria from AbbVie, Astellas, Bristol Myers Squibb, Daiichi Sankyo, Jazz, Janssen, Novartis, Otsuka, Pfizer, and Servier; institutional research funding from AbbVie, Astellas, Novartis, and Pfizer. **K.D.**: honoraria from Novartis, Jazz Pharmaceuticals, and AbbVie; has participated in Ad Boards from Novartis, Bristol Myers Squibb, Jazz Pharmaceuticals, and AbbVie; research funding from Novartis, Astellas, Agios, Bristol Myers Squibb, and Kronos. **M.H.**: honoraria from Astellas, Daiichi Sankyo, Janssen, Miltenyi, Otsuka, Qiagen, and Servier and has participated in Ad Boards from AbbVie, AvenCell, Ascentage Pharma, Bristol Myers Squibb, Janssen, Jazz Pharmaceuticals, LabDelbert, Novartis, Pfizer, and Servier; research funding from AbbVie, Bayer Pharma AG, Jazz Pharmaceuticals, Glycostem, Karyopharm, PinotBio, Servier, and Toray. **T.H.**: current employment at Munich Leukemia Laboratory, with part ownership. **J.M.H.‐R.**: honoraria from Bristol Myers Squibb, Pfizer, Amgen, Celgene, GSK, and Novartis; advisory role for Bristol Myers Squibb, Pfizer, Amgen, Celgene, and Novartis; research funding from GSK and Pfizer. **B.J.P.H.**: honoraria from Pfizer, Bristol Myers Squibb, and Novartis. **G.O.**: honoraria from AbbVie, Jazz Pharmaceuticals, Astellas, Gilead, Bristol Myers Squibb, Servier, and Roche. **H.D.**: advisory role for AbbVie, Agios, Amgen, Astellas, AstraZeneca, BERLIN‐CHEMIE, Bristol Myers Squibb, Celgene, GEMoaB, Gilead Sciences, Janssen, Jazz Pharmaceuticals, Novartis, and Syndax; research funding from AbbVie, Agios, Amgen, Astellas, Bristol Myers Squibb, Jazz Pharmaceuticals, Kronos‐Bio, and Novartis. **L.B.**: honoraria from AbbVie, Amgen, Astellas, Bristol Myers Squibb, Celgene, Daiichi Sankyo, Gilead, Hexal, Janssen, Jazz Pharmaceuticals, Menarini, Novartis, Otsuka, Pfizer, Roche, and Sanofi; research funding from Bayer and Jazz Pharmaceuticals.

## FUNDING

HARMONY and HARMONY PLUS are funded through the Innovative Medicines Initiative (IMI), Europe's largest public–private initiative aiming to speed up the development of better and safer medicines for patients. Funding is received from the IMI 2 Joint Undertaking and is listed under the grant agreement for HARMONY No. 116026 and the grant agreement for HARMONY PLUS No. 945406. This Joint Undertaking receives support from the European Union's Horizon 2020 Research and Innovation Program and the European Federation of Pharmaceutical Industries and Associations (EFPIA). This study presents results from the AML workgroup in HARMONY.

A.H.‐S. was supported by Contrato Río Hortega CM23/00101 (ISCIII). A.T.T. received additional support from the Deutsche Forschungsgemeinschaft (DFG) grant FU 356/12‐1 (ATT). R.M. was supported by the Mildred‐Scheel‐Postdoctoral‐Program from Deutsche Krebshilfe (grant no. 70115737).

## Supporting information

Supporting Information.

## Data Availability

After the publication of this article, data collected for this analysis and related documents will be made available to others upon reasonably justified request, which needs to be written and addressed to the attention of the corresponding author Dr. Lars Bullinger at the following e‐mail address: lars.bullinger@charite.de. The HARMONY Alliance, via the corresponding author Dr. Lars Bullinger, is responsible to evaluate and eventually accept or refuse every request to disclose data and their related documents, in compliance with the ethical approval conditions, in compliance with applicable laws and regulations, and in conformance with the agreements in place with the involved subjects, the participating institutions, and all the other parties directly or indirectly involved in the participation, conduct, development, management, and evaluation of this analysis.
