# Supplementary material for: Machine learning provides individualized prediction of outcomes after first complete remission without allo‐HSCT consolidation in adult acute myeloid leukemia—A HARMONY study
Source: Hemasphere. 2026 Aug 30;10(9):e70461. doi: 10.1002/hem3.70461 (PMC13526294; doi:10.1002/hem3.70461)
Supplement: Supplementary file 1 [file HEM3-10-e70461-s001.docx]

**Supplementary Appendix**

to

Machine Learning Provides Individualized Prediction of Outcomes after First Complete Remission without Allo-HSCT Consolidation in Adult Acute Myeloid Leukemia – A HARMONY Study

by Hernández-Sánchez et al.

**Table of contents**

[Supplementary methods	2](#_Toc232684852)

[References	8](#_Toc232684853)

[Supplementary figures	9](#_Toc232684854)

[Figure S1. Overall survival of relapsed patients, from the relapse date, in the training cohort.	9](#_Toc232684855)

[Figure S2. Overall survival of relapsed patients, from the relapse date and stratified by ELN2022 risk groups, in the training cohort.	9](#_Toc232684856)

[Figure S3. Overall survival of relapsed patients from the date of relapse, stratified by whether patients underwent allo-HSCT and estimated with the Simon–Makuch method (clock-back correction).	10](#_Toc232684857)

[Figure S4. Density plot of predicted 5-year CIR according to HARMONY machine learning model, stratified by ELN2022 risk classification categories, in the training cohort.	11](#_Toc232684858)

[Figure S5. Calibration plot of the HARMONY machine learning model between predicted and observed outcomes at 5 years, divided by quintiles, in the training cohort (A, C, E) and the external validation cohort (B, D, F).	12](#_Toc232684859)

[Figure S6. Predictive performance of the simplified HARMONY ML model (cytogenetic abnormalities and gene mutations only, without taking into account patient age, sex and AML type) compared to ELN2022 risk classification, measured by AUC(t) at 1, 2, 3, 4 and 5 years	13](#_Toc232684860)

[Figure S7. Density plot of predicted 5-year CIR according to HARMONY machine learning model, stratified by ELN2022 risk classification categories, in the external validation cohort.	14](#_Toc232684861)

[Supplementary tables	15](#_Toc232684862)

[Table S1. Distribution of genomic aberrations included in the machine learning model, in the training cohort.	15](#_Toc232684863)

[Table S2. Comparison of predictive performance, measured by the area under the receiver operating curve (AUC(t)) of HARMONY machine learning model, based on Bayesian Additive Regression Trees (BART), compared to European LeukemiaNet (ELN) 2022 risk classification in the training cohort.	17](#_Toc232684864)

[Table S3. Distribution of genomic aberrations included in the machine learning model, in the external validation cohort.	21](#_Toc232684865)

[Table S4. Comparison of predictive performance, measured by the area under the receiver operating curve (AUC(t)) of HARMONY machine learning model, based on Bayesian Additive Regression Trees (BART), compared to European LeukemiaNet (ELN) 2022 risk classification in the external validation cohort.	23](#_Toc232684866)

[Table S5. Comparison of predictive performance, measured by concordance index (C-index) of HARMONY machine learning model, compared to European LeukemiaNet (ELN) 2022 risk classification	23](#_Toc232684867)

# Supplementary methods

This section outlines the process of data extraction and statistical procedures for development of the machine learning model using Bayesian Additive Regression Trees (BART).

**HARMONY database**

Patient data uploaded to the HARMONY platform undergoes a thorough double-broker pseudonymization process in compliance to the General Data Protection Regulation (GDPR). The HARMONY platform harmonizes and transforms data from various sources using the Observational Medical Outcomes Partnership (OMOP) Common Data Model standard^1^. Data is integrated into a global “data lake” containing information on patients with different diseases and separate workspaces are created with the required data for each research project. Access to the data is conducted through R language (version 3.6.3) via Apache Zeppelin notebooks, with a dedicated Virtual Private Network (VPN) to ensure data security.

**Data pre-processing**

The initial step consisted in extracting relevant data for analysis from the OMOP database. In this database, concepts are represented by numeric codes stored in various tables, accessible through Structured Query Language (SQL) queries. The extracted information was transformed into a tabular structure where rows represent patients and columns represent the different patient associated concepts, such as age at diagnosis, gender, gene mutations, International System for Human Cytogenomic Nomenclature (ISCN) karyotype formula^2^, AML type, allogeneic hematopoietic stem cell transplantation (allo-HSCT) date, first complete remission (CR1) date, relapse date and death or last follow-up date, among others.

This initial step is time-consuming, as it requires rigorous quality controls and pre-processing activities, such as developing a parser to decode chromosomal abnormalities from ISCN karyotype formula or calculating the European LeukemiaNet (ELN) 2022 risk classification for each patient^3^.

A similar process was applied to the external validation cohort with the data published by Tazi *et al*.^4^ which is publicly available from a [GitHub repository](https://github.com/papaemmelab/Tazi_NatureC_AML). As the data source was not encoded in OMOP, it required combining different files and adapting the information to a similar format to that extracted from the HARMONY database. In addition, ELN2022 risk classification had to be calculated, as the original publication and source files used the previous ELN2017 classification^5^.

Following data preparation, a cohort of 2,550 patients was selected for model training, based on the inclusion and exclusion criteria mentioned in the main manuscript (**Figure 1**). The external validation cohort of 714 patients was also selected using similar criteria, with the exception that patients who received targeted therapies in addition to intensive chemotherapy regimens were also included.

**Missing data imputation**

A single imputation process was applied to the HARMONY dataset to address missing data. Of note, this step was not necessary for the external validation cohort, as it did not contain missing values for the variables used in the model.

Imputation involves replacing missing values with estimates derived from statistical relationships among observed variables. In this study, we initially applied multiple imputation using the mice (Multivariate Imputation by Chained Equations) package with a Random Forest prediction method, selected for its ability to capture nonlinear relationships and complex interactions between variables.

However, unlike parametric models such as Cox regression, which allow the pooling of coefficients across models fitted from different imputed datasets using Rubin’s rules^6^, BART does not allow construction of a single pooled model. Consequently, predictions must be generated separately from each model corresponding to each imputed dataset and then averaged, resulting in considerable computational overhead. After studying alternatives, we found that **s**imple imputation yielded negligible differences in predictions and final AUC metrics. We therefore opted for simple imputation to optimize computational efficiency.

Patterns of missingness in binary variables indicating gene mutation status were explored using *mice*, *naniar* and *Hmisc* packages. In the HARMONY dataset, missing data resulted from the use of distinct next generation sequencing (NGS) myeloid panels across data sources. Variables with a missingness rate exceeding 20%, reflecting mutations not evaluated in a significant proportion of the panels, were excluded from the imputation process. The remaining variables subjected to imputation included bZIP in-frame mutated *CEBPA* (17.6%), other *CEBPA* mutations (17.6%), *FLT3* tyrosine kinase domain mutation (*FLT3*-TKD, 5.5%), *WT1* (2.5%), *STAG2* (2.5%), *PTPN11* (2.5%), *BCOR* (2.5%) and *SRSF2* (0.2%). According to Rubin’s classification of missing data, the data were considered missing at random (MAR), as the probability of missingness depended on observed variables (e.g., the panel used) but not on the variable itself^6^. Consequently, the aforementioned imputation process was deemed appropriate.

**BART models**

Once the training dataset was prepared, survival models were developed for predicting overall survival (OS), relapse-free survival (RFS) and cumulative incidence of relapse (CIR). We selected Bayesian Additive Regression Trees^7–9^ as our preferred modelling approach for several reasons:

a) Bayesian approach: BART is a nonlinear model that leverages the advantages of the Bayesian paradigm. It can provide not only predictions but also measures of uncertainty, including confidence intervals for the predictions. In our opinion this feature is crucial when predicting patient outcomes.
b) Managing numerous variables: BART can manage a large number of covariates and inherently incorporates interactions between them without requiring explicit specification in the model.
c) Non-parametric flexibility: As a non-parametric model, BART does not require certain assumptions about the underlying data distribution of variables, enhancing its versatility and ease of use.
d) Computational efficiency: The BART library supports parallel processing, which can significantly reduce training time when adequate computational resources are available.

The original formulation of the BART model was developed by Chipman *et al ^10^*. In simple terms, BART can be regarded as a sum-of-trees ensemble^11^. The predictive function is the sum of numerous regression trees, where the first tree explains a small proportion of the variation and each subsequent tree added to the sum attempts to explain the residuals. BART consists of a set of priors for the structure and leaf parameters, along with a likelihood for the data in the terminal nodes. In order to prevent overfitting, the trees are constrained to be small, acting as “weak” learners. A regularization prior limits the size and fit of each tree, ensuring that each one only contributes to a small proportion of the overall fit. Posterior inference in BART models is typically conducted via Markov Chain Monte Carlo (MCMC), where a Metropolis-within-Gibbs sampler is used to generate samples from the posterior distribution^11^. The BART library also supports competing risk as an extension of BART survival analysis.

While BART is a “black box” machine learning model where non-linear covariate functions are difficult to interpret, other popular black-box models such as neural networks, random forests or gradient boosting have demonstrated good precision and are widely used. To enhance interpretability, we provide a sensitivity analysis for each simulation. This analysis identifies the ten most influential variables and illustrates their impact on predictions alongside their respective confidence intervals.

**Model validation**

The predictive performance of the BART model was evaluated through 10-fold cross-validation. The time-dependent Area Under receiver operating Curve (AUC(t)) was calculated at 1, 2, 3, 4 and 5 years for OS, RFS and CIR using timeROC library^12,13^. Furthermore, BART’s performance was compared against the ELN2022 risk classification using the method described by Blanche *et al*^12,13^ to calculate corresponding p-values (**Tables S2 and S4)**.

Moreover, we also evaluated the predictive performance of the final model (trained with the HARMONY dataset of 2,550 patients) in the external validation cohort (n=714), comparing time-dependent AUC at 1, 2, 3, 4 and 5 years between the BART model and ELN2022 risk classification.

**Online tool for individualized prediction of outcomes**

We developed a web-based interface to provide individualized outcome estimations for OS, RFS and CIR based on our BART models (<https://geneticsoncohematology.com/AML_Outcome_Predictor/>). Future versions will support batch processing for multiple patients. Designed using the Shiny library, the responsive interface adapts to various screen sizes and generates predictions using five input variables: age, gender, AML type, cytogenetic abnormalities and gene mutations (**Figure 5**). As noted, BART Bayesian models estimate a probability distribution for each patient profile rather than a single estimation. To ensure a robust and stable prediction, the tool performs 1,000 draws per model. From these samples, the 5th and 95th percentiles are calculated and displayed as confidence intervals in the graphical representation. The graph also includes reference survival curves based on ELN2022 risk categories. Finally, the interface features a 5-year CIR sensitivity analysis, illustrating how outcome predictions might vary if the patient had different genomic alterations.

# References

1.	Belenkaya R, Gurley MJ, Golozar A, et al. Extending the OMOP Common Data Model and Standardized Vocabularies to Support Observational Cancer Research. *JCO Clin Cancer Inform*. 2021;5:12-20. doi:10.1200/cci.20.00079

2.	McGowan-Jordan J, Hastings RJ, Moore S, eds. *ISCN 2020: An International System for Human Cytogenomic Nomenclature (2020)*. S. Karger; 2020. doi:10.1159/isbn.978-3-318-06867-2

3.	Döhner H, Wei AH, Appelbaum FR, et al. Diagnosis and management of AML in adults: 2022 recommendations from an international expert panel on behalf of the ELN. *Blood*. 2022;140(12):1345-1377. doi:10.1182/blood.2022016867

4.	Tazi Y, Arango-Ossa JE, Zhou Y, et al. Unified classification and risk-stratification in Acute Myeloid Leukemia. *Nat Commun*. 2022;13(1):4622. doi:10.1038/s41467-022-32103-8

5.	Döhner H, Estey E, Grimwade D, et al. Diagnosis and management of AML in adults: 2017 ELN recommendations from an  international expert panel. *Blood*. 2017;129(4):424-447. doi:10.1182/blood-2016-08-733196

6.	van Buuren S. *Flexible Imputation of Missing Data, Second Edition*. Chapman and Hall/CRC; 2018. doi:10.1201/9780429492259

7.	Sparapani R, Spanbauer C, McCulloch R. Nonparametric Machine Learning and Efficient Computation with Bayesian Additive Regression Trees: The BART R Package. *J Stat Softw*. 2021;97(1):1-66. doi:10.18637/JSS.V097.I01

8.	Sparapani R, Logan BR, McCulloch RE, Laud PW. Nonparametric competing risks analysis using Bayesian Additive Regression Trees. *Stat Methods Med Res*. 2020;29(1):57-77. doi:10.1177/0962280218822140

9.	Sparapani RA, Logan BR, McCulloch RE, Laud PW. Nonparametric survival analysis using Bayesian Additive Regression Trees (BART). *Stat Med*. 2016;35(16):2741-2753. doi:10.1002/SIM.6893

10.	Chipman HA, George EI, McCulloch RE. BART: Bayesian additive regression trees. *Annals of Applied Statistics*. 2010;4(1):266-298. doi:10.1214/09-AOAS285

11.	Hill J, Linero A, Murray J. Bayesian Additive Regression Trees: A Review and Look Forward. *Annu Rev Stat Appl*. 2020;7:251-278. doi:10.1146/annurev-statistics-031219-041110

12.	Blanche P, Dartigues JF, Jacqmin-Gadda H. Estimating and comparing time-dependent areas under receiver operating characteristic curves for censored event times with competing risks. *Stat Med*. 2013;32(30):5381-5397. doi:10.1002/sim.5958

13.	Kamarudin AN, Cox T, Kolamunnage-Dona R. Time-dependent ROC curve analysis in medical research: current methods and applications. *BMC Med Res Methodol*. 2017;17(1):53. doi:10.1186/S12874-017-0332-6

14.	Bernasconi DP, Rebora P, Iacobelli S, Valsecchi MG, Antolini L. Survival probabilities with time-dependent treatment indicator: Quantities and non-parametric estimators. *Stat Med*. 2016;35(7):1032-1048. doi:10.1002/sim.6765

# Supplementary figures

## Figure S1. Overall survival of relapsed patients, from the relapse date, in the training cohort.

## Figure S2. Overall survival of relapsed patients, from the relapse date and stratified by ELN2022 risk groups, in the training cohort.

## Figure S3. Overall survival of relapsed patients from the date of relapse, stratified by whether patients underwent allo-HSCT and estimated with the Simon–Makuch method^14^ (clock-back correction).

## Figure S4. Density plot of predicted 5-year CIR according to HARMONY machine learning model, stratified by ELN2022 risk classification categories, in the training cohort.

## Figure S5. Calibration plot of the HARMONY machine learning model between predicted and observed outcomes at 5 years, divided by quintiles, in the training cohort (A, C, E) and the external validation cohort (B, D, F).

## Figure S6. Predictive performance of the simplified HARMONY ML model (cytogenetic abnormalities and gene mutations only, without taking into account patient age, sex and AML type) compared to ELN2022 risk classification, measured by AUC(t) at 1, 2, 3, 4 and 5 years (A, B) Overall survival in training and external validation cohort, respectively. (C, D) Relapse-free survival in training and external validation cohort, respectively. (E, F) Cumulative incidence of relapse in training and validation cohort, respectively

## Figure S7. Density plot of predicted 5-year CIR according to HARMONY machine learning model, stratified by ELN2022 risk classification categories, in the external validation cohort.

# Supplementary tables

## Table S1. Distribution of genomic aberrations included in the machine learning model, in the training cohort.

| **Genomic aberration / gene mutation** | **Number of patients** | **% of cohort** | **% of missings (imputed)** |
| --- | --- | --- | --- |
| Normal karyotype | 1327 | 52.04 | 0 |
| *NPM1* | 921 | 36.12 | 0 |
| *DNMT3A* | 671 | 26.31 | 0 |
| *NRAS* | 502 | 19.69 | 0 |
| *FLT3*-ITD | 484 | 18.98 | 0 |
| t(8;21) | 365 | 14.31 | 0 |
| *TET2* | 345 | 13.53 | 0 |
| *IDH2* | 302 | 11.84 | 0 |
| *FLT3*-TKD | 278 | 10.90 | 5.5 |
| *KIT* | 213 | 8.35 | 0 |
| *PTPN11* | 213 | 8.35 | 2.5 |
| *RUNX1* | 204 | 8.00 | 0 |
| inv(16)/t(16;16) | 202 | 7.92 | 0 |
| *WT1* | 193 | 7.57 | 2.5 |
| *IDH1* | 190 | 7.45 | 0 |
| bZIP in-frame mutated *CEBPA* | 188 | 7.37 | 17.6* |
| *ASXL1* | 177 | 6.94 | 0 |
| -Y | 176 | 6.90 | 0 |
| +8 | 167 | 6.55 | 0 |
| *KRAS* | 149 | 5.84 | 0 |
| Complex karyotype | 140 | 5.49 | 0 |
| *SRSF2* | 138 | 5.41 | 0.2 |
| *TP53* | 117 | 4.59 | 0 |
| Monosomal karyotype | 111 | 4.35 | 0 |
| Marker chromosome | 96 | 3.76 | 0 |
| *EZH2* | 94 | 3.69 | 0 |
| *STAG2* | 94 | 3.69 | 2.5 |
| *BCOR* | 85 | 3.33 | 2.5 |
| -5/del(5q) | 85 | 3.33 | 0 |
| del(9q) | 83 | 3.25 | 0 |
| -X | 71 | 2.78 | 0 |
| -17/abn(17p) | 63 | 2.47 | 0 |
| -7 | 62 | 2.43 | 0 |
| Other *CEBPA* mutations | 60 | 2.35 | 17.6 |
| +22 | 55 | 2.16 | 0 |
| t(v;11q23.3) | 53 | 2.08 | 0 |
| *U2AF1* | 53 | 2.08 | 0 |
| -12/abn(12p) | 50 | 1.96 | 0 |
| +21 | 50 | 1.96 | 0 |
| *JAK2* | 49 | 1.92 | 0 |
| del(7q) | 48 | 1.88 | 0 |
| *SF3B1* | 45 | 1.76 | 0 |
| -17 | 43 | 1.69 | 0 |
| -18 | 41 | 1.61 | 0 |
| -5 | 36 | 1.41 | 0 |
| +13 | 27 | 1.06 | 0 |
| -16 | 25 | 0.98 | 0 |
| +4 | 24 | 0.94 | 0 |
| -21 | 23 | 0.90 | 0 |
| t(9;11) | 21 | 0.82 | 0 |
| -13 | 21 | 0.82 | 0 |
| -20 | 21 | 0.82 | 0 |
| *ZRSR2* | 21 | 0.82 | 0 |
| -12 | 20 | 0.78 | 0 |
| +6 | 19 | 0.75 | 0 |
| +11 | 18 | 0.71 | 0 |
| del(11q) | 16 | 0.63 | 0 |
| -3 | 15 | 0.59 | 0 |
| +19 | 15 | 0.59 | 0 |
| del(13q) | 14 | 0.55 | 0 |
| del(20q) | 14 | 0.55 | 0 |
| -22 | 14 | 0.55 | 0 |
| add(21q) | 13 | 0.51 | 0 |
| -19 | 13 | 0.51 | 0 |
| +10 | 13 | 0.51 | 0 |
| add(11q) | 12 | 0.47 | 0 |
| add(7q) | 12 | 0.47 | 0 |
| -8 | 12 | 0.47 | 0 |
| +1 | 12 | 0.47 | 0 |
| +15 | 11 | 0.43 | 0 |
| t(6;9) | 11 | 0.43 | 0 |
| add(1q) | 10 | 0.39 | 0 |
| del(3q) | 10 | 0.39 | 0 |
| *MECOM*r | 10 | 0.39 | 0 |
| -11 | 10 | 0.39 | 0 |
| +9 | 10 | 0.39 | 0 |

* 449/2550 patients (17.6%) had missing CEBPA bZIP status, which was imputed as follows. In 311 cases the gene was not covered by the NGS panel—predominantly a multicenter cohort of patients with t(8;21)(q22;q22.1)/RUNX1::RUNX1T1 AML, in which CEBPA bZIP mutations are exceedingly rare—and only 2 of these were imputed as CEBPA bZIP-positive. In the remaining cases, 90 were reported as biallelic CEBPA and 65 (72%) were imputed as CEBPA bZIP and 48 cases were reported as monoallelic CEBPA and 12 (25%) were imputed as CEBPA bZIP.

## Table S2. Comparison of predictive performance, measured by the area under the receiver operating curve (AUC(t)) of HARMONY machine learning model, based on Bayesian Additive Regression Trees (BART), compared to European LeukemiaNet (ELN) 2022 risk classification in the training cohort.

10-fold cross validation was performed for each time point (1, 2, 3, 4 and 5 years) and outcome type (overall survival [OS], relapse-free survival [RFS] and cumulative incidence of relapse [CIR]).

| **Outcome type** | **Time (years)** | **AUC(t) (BART)** | **Standard error (BART)** | **AUC(t) (ELN2022)** | **Standard error (ELN2022)** | **p-value** |
| --- | --- | --- | --- | --- | --- | --- |
| OS | 1 | 0.71366 | 0.03623 | 0.68061 | 0.03489 | 0.1212 |
| OS | 2 | 0.7354 | 0.03287 | 0.6856 | 0.03303 | 0.0108 |
| OS | 3 | 0.7445 | 0.03212 | 0.68456 | 0.03226 | 0.0022 |
| OS | 4 | 0.72291 | 0.03373 | 0.67366 | 0.03297 | 0.0201 |
| OS | 5 | 0.7019 | 0.03658 | 0.65566 | 0.03567 | 0.0471 |
| OS | 1 | 0.76339 | 0.0364 | 0.66499 | 0.03646 | <0.0001 |
| OS | 2 | 0.73169 | 0.03377 | 0.66985 | 0.03252 | 0.0032 |
| OS | 3 | 0.74666 | 0.03175 | 0.68555 | 0.03106 | 0.0041 |
| OS | 4 | 0.72386 | 0.03366 | 0.66785 | 0.0323 | 0.0114 |
| OS | 5 | 0.72464 | 0.03494 | 0.66783 | 0.03461 | 0.0211 |
| OS | 1 | 0.81867 | 0.0325 | 0.73388 | 0.03473 | 0.0004 |
| OS | 2 | 0.75554 | 0.03192 | 0.69019 | 0.0326 | 0.0013 |
| OS | 3 | 0.75188 | 0.0315 | 0.71804 | 0.03085 | 0.0863 |
| OS | 4 | 0.72847 | 0.03358 | 0.70343 | 0.03158 | 0.2217 |
| OS | 5 | 0.71674 | 0.03634 | 0.70411 | 0.03287 | 0.5773 |
| OS | 1 | 0.75881 | 0.03224 | 0.69418 | 0.03393 | 0.0093 |
| OS | 2 | 0.72497 | 0.03224 | 0.70269 | 0.03159 | 0.3494 |
| OS | 3 | 0.73435 | 0.03229 | 0.70693 | 0.03141 | 0.2286 |
| OS | 4 | 0.75185 | 0.03206 | 0.72215 | 0.031 | 0.1915 |
| OS | 5 | 0.72885 | 0.03493 | 0.70643 | 0.03301 | 0.3523 |
| OS | 1 | 0.80743 | 0.03032 | 0.75155 | 0.03153 | 0.0073 |
| OS | 2 | 0.79136 | 0.03017 | 0.7623 | 0.02867 | 0.1079 |
| OS | 3 | 0.77259 | 0.03052 | 0.74638 | 0.02908 | 0.1481 |
| OS | 4 | 0.77034 | 0.0312 | 0.73775 | 0.03058 | 0.0846 |
| OS | 5 | 0.7685 | 0.03275 | 0.73643 | 0.03194 | 0.101 |
| OS | 1 | 0.7385 | 0.03749 | 0.68117 | 0.03755 | 0.0157 |
| OS | 2 | 0.75937 | 0.03175 | 0.71736 | 0.03163 | 0.0357 |
| OS | 3 | 0.7747 | 0.03107 | 0.7121 | 0.03133 | 0.0026 |
| OS | 4 | 0.78155 | 0.03034 | 0.71453 | 0.03124 | 0.0013 |
| OS | 5 | 0.79122 | 0.03041 | 0.71546 | 0.03226 | 0.0004 |
| OS | 1 | 0.75642 | 0.03404 | 0.69501 | 0.03643 | 0.0206 |
| OS | 2 | 0.76049 | 0.03181 | 0.68264 | 0.03319 | 0.0005 |
| OS | 3 | 0.77013 | 0.03016 | 0.70401 | 0.03102 | 0.0016 |
| OS | 4 | 0.77271 | 0.03087 | 0.72913 | 0.02988 | 0.0373 |
| OS | 5 | 0.76935 | 0.03242 | 0.72474 | 0.03059 | 0.0447 |
| OS | 1 | 0.68817 | 0.03801 | 0.61972 | 0.03666 | 0.0044 |
| OS | 2 | 0.72465 | 0.03342 | 0.68655 | 0.03214 | 0.0742 |
| OS | 3 | 0.69765 | 0.03388 | 0.6758 | 0.03203 | 0.3086 |
| OS | 4 | 0.69396 | 0.0352 | 0.69383 | 0.03238 | 0.9955 |
| OS | 5 | 0.68728 | 0.03675 | 0.68362 | 0.03408 | 0.8791 |
| OS | 1 | 0.80164 | 0.03229 | 0.73958 | 0.03414 | 0.0068 |
| OS | 2 | 0.76955 | 0.03234 | 0.72019 | 0.03098 | 0.0167 |
| OS | 3 | 0.7639 | 0.03236 | 0.71556 | 0.03054 | 0.0198 |
| OS | 4 | 0.77112 | 0.03189 | 0.72231 | 0.03029 | 0.0198 |
| OS | 5 | 0.76898 | 0.03286 | 0.72369 | 0.03104 | 0.0344 |
| OS | 1 | 0.73085 | 0.03679 | 0.67883 | 0.03583 | 0.0076 |
| OS | 2 | 0.75913 | 0.03093 | 0.68813 | 0.03249 | <0.0001 |
| OS | 3 | 0.76139 | 0.0305 | 0.69582 | 0.03179 | 0.0001 |
| OS | 4 | 0.75229 | 0.0316 | 0.69172 | 0.03181 | 0.0006 |
| OS | 5 | 0.75384 | 0.03185 | 0.68283 | 0.03193 | 0.0001 |
| RFS | 1 | 0.72794 | 0.03264 | 0.69577 | 0.0316 | 0.0908 |
| RFS | 2 | 0.70551 | 0.0328 | 0.68237 | 0.03069 | 0.2197 |
| RFS | 3 | 0.70543 | 0.03303 | 0.6872 | 0.03075 | 0.3515 |
| RFS | 4 | 0.7106 | 0.03375 | 0.68551 | 0.03192 | 0.2214 |
| RFS | 5 | 0.71158 | 0.03486 | 0.68286 | 0.03306 | 0.1984 |
| RFS | 1 | 0.68917 | 0.03449 | 0.65783 | 0.03225 | 0.1315 |
| RFS | 2 | 0.70546 | 0.03287 | 0.66753 | 0.03095 | 0.0708 |
| RFS | 3 | 0.69725 | 0.03385 | 0.66147 | 0.03169 | 0.1112 |
| RFS | 4 | 0.71877 | 0.03357 | 0.67978 | 0.03159 | 0.0709 |
| RFS | 5 | 0.72418 | 0.03515 | 0.68401 | 0.03261 | 0.0805 |
| RFS | 1 | 0.78359 | 0.02914 | 0.71644 | 0.02993 | 0.0001 |
| RFS | 2 | 0.75334 | 0.03031 | 0.68286 | 0.02992 | 0.0001 |
| RFS | 3 | 0.76094 | 0.03062 | 0.68368 | 0.03019 | 0.0001 |
| RFS | 4 | 0.74325 | 0.0328 | 0.66648 | 0.0322 | 0.0002 |
| RFS | 5 | 0.74457 | 0.03515 | 0.66053 | 0.03508 | 0.0002 |
| RFS | 1 | 0.79223 | 0.02881 | 0.72626 | 0.03021 | 0.0001 |
| RFS | 2 | 0.79285 | 0.02773 | 0.72196 | 0.02951 | <0.0001 |
| RFS | 3 | 0.78537 | 0.02884 | 0.72671 | 0.02934 | 0.0006 |
| RFS | 4 | 0.79166 | 0.02909 | 0.73568 | 0.02918 | 0.002 |
| RFS | 5 | 0.77502 | 0.03191 | 0.72337 | 0.0309 | 0.0069 |
| RFS | 1 | 0.76726 | 0.02977 | 0.73976 | 0.02944 | 0.16 |
| RFS | 2 | 0.76471 | 0.03016 | 0.74742 | 0.02775 | 0.3666 |
| RFS | 3 | 0.77028 | 0.03057 | 0.74715 | 0.02766 | 0.2318 |
| RFS | 4 | 0.77653 | 0.0314 | 0.74987 | 0.02776 | 0.1833 |
| RFS | 5 | 0.77448 | 0.03459 | 0.73964 | 0.03048 | 0.1025 |
| RFS | 1 | 0.71616 | 0.03228 | 0.7009 | 0.03034 | 0.3863 |
| RFS | 2 | 0.73801 | 0.03075 | 0.71235 | 0.02853 | 0.1579 |
| RFS | 3 | 0.75281 | 0.03053 | 0.72457 | 0.02804 | 0.1307 |
| RFS | 4 | 0.73253 | 0.03323 | 0.71141 | 0.02976 | 0.2929 |
| RFS | 5 | 0.7144 | 0.036 | 0.69458 | 0.03187 | 0.3659 |
| RFS | 1 | 0.73784 | 0.03182 | 0.67691 | 0.03185 | 0.0017 |
| RFS | 2 | 0.77625 | 0.02974 | 0.67761 | 0.02983 | <0.0001 |
| RFS | 3 | 0.79141 | 0.02973 | 0.69566 | 0.02901 | <0.0001 |
| RFS | 4 | 0.78989 | 0.0306 | 0.69999 | 0.02889 | <0.0001 |
| RFS | 5 | 0.79499 | 0.03192 | 0.69687 | 0.0304 | <0.0001 |
| RFS | 1 | 0.77728 | 0.0295 | 0.72823 | 0.03 | 0.0046 |
| RFS | 2 | 0.7961 | 0.02823 | 0.74095 | 0.02867 | 0.0016 |
| RFS | 3 | 0.80178 | 0.0283 | 0.74011 | 0.02873 | 0.0004 |
| RFS | 4 | 0.80982 | 0.02883 | 0.74282 | 0.02951 | 0.0001 |
| RFS | 5 | 0.80896 | 0.02991 | 0.74971 | 0.02956 | 0.0013 |
| RFS | 1 | 0.78708 | 0.02975 | 0.73886 | 0.02953 | 0.0069 |
| RFS | 2 | 0.77078 | 0.02904 | 0.71999 | 0.02827 | 0.0065 |
| RFS | 3 | 0.78831 | 0.02798 | 0.73104 | 0.02741 | 0.0029 |
| RFS | 4 | 0.78306 | 0.0294 | 0.73765 | 0.02732 | 0.0256 |
| RFS | 5 | 0.77918 | 0.03162 | 0.72543 | 0.02861 | 0.0166 |
| RFS | 1 | 0.70356 | 0.03411 | 0.68004 | 0.03148 | 0.2315 |
| RFS | 2 | 0.67104 | 0.03406 | 0.67111 | 0.03052 | 0.9968 |
| RFS | 3 | 0.70041 | 0.03334 | 0.69117 | 0.03005 | 0.6093 |
| RFS | 4 | 0.7126 | 0.03382 | 0.70816 | 0.02978 | 0.8069 |
| RFS | 5 | 0.69619 | 0.03666 | 0.69396 | 0.0314 | 0.9088 |
| CIR | 1 | 0.73055 | 0.03456 | 0.68074 | 0.03237 | 0.015 |
| CIR | 2 | 0.69113 | 0.03481 | 0.65453 | 0.03147 | 0.0762 |
| CIR | 3 | 0.67957 | 0.03579 | 0.65857 | 0.03147 | 0.3213 |
| CIR | 4 | 0.68479 | 0.03691 | 0.66604 | 0.0319 | 0.3936 |
| CIR | 5 | 0.6823 | 0.03911 | 0.68219 | 0.03211 | 0.9963 |
| CIR | 1 | 0.76202 | 0.03312 | 0.73207 | 0.03253 | 0.1549 |
| CIR | 2 | 0.73521 | 0.0329 | 0.72796 | 0.03046 | 0.7182 |
| CIR | 3 | 0.73977 | 0.03273 | 0.72796 | 0.03036 | 0.5654 |
| CIR | 4 | 0.73465 | 0.03361 | 0.73496 | 0.03029 | 0.9879 |
| CIR | 5 | 0.70226 | 0.03702 | 0.70665 | 0.03334 | 0.8547 |
| CIR | 1 | 0.75676 | 0.03349 | 0.72059 | 0.03198 | 0.1005 |
| CIR | 2 | 0.75925 | 0.032 | 0.72631 | 0.03044 | 0.1056 |
| CIR | 3 | 0.75284 | 0.03298 | 0.72261 | 0.0309 | 0.1531 |
| CIR | 4 | 0.75297 | 0.03422 | 0.72558 | 0.03176 | 0.2031 |
| CIR | 5 | 0.77196 | 0.03474 | 0.73771 | 0.03214 | 0.1294 |
| CIR | 1 | 0.73603 | 0.03383 | 0.6831 | 0.03372 | 0.02 |
| CIR | 2 | 0.71732 | 0.03437 | 0.67311 | 0.03272 | 0.0474 |
| CIR | 3 | 0.73503 | 0.03531 | 0.68763 | 0.03316 | 0.0433 |
| CIR | 4 | 0.73241 | 0.03731 | 0.68918 | 0.03417 | 0.0555 |
| CIR | 5 | 0.72293 | 0.03913 | 0.68837 | 0.03489 | 0.1327 |
| CIR | 1 | 0.75716 | 0.03452 | 0.73191 | 0.03278 | 0.2479 |
| CIR | 2 | 0.75873 | 0.03254 | 0.7136 | 0.03093 | 0.0337 |
| CIR | 3 | 0.77403 | 0.03218 | 0.72766 | 0.02992 | 0.0316 |
| CIR | 4 | 0.7831 | 0.03189 | 0.72939 | 0.03015 | 0.0134 |
| CIR | 5 | 0.76733 | 0.03451 | 0.71697 | 0.03166 | 0.0298 |
| CIR | 1 | 0.74787 | 0.03432 | 0.70176 | 0.03472 | 0.042 |
| CIR | 2 | 0.76588 | 0.03198 | 0.72587 | 0.03178 | 0.0421 |
| CIR | 3 | 0.76544 | 0.03229 | 0.7238 | 0.0315 | 0.0396 |
| CIR | 4 | 0.76357 | 0.03432 | 0.7382 | 0.03152 | 0.2289 |
| CIR | 5 | 0.75916 | 0.03795 | 0.73232 | 0.03308 | 0.2521 |
| CIR | 1 | 0.75731 | 0.03233 | 0.69847 | 0.0322 | 0.0047 |
| CIR | 2 | 0.75554 | 0.0321 | 0.67533 | 0.03104 | 0.0001 |
| CIR | 3 | 0.74761 | 0.03306 | 0.66965 | 0.03124 | 0.0003 |
| CIR | 4 | 0.76195 | 0.0341 | 0.67651 | 0.03164 | 0.0002 |
| CIR | 5 | 0.76207 | 0.03722 | 0.67453 | 0.03281 | 0.0002 |
| CIR | 1 | 0.76186 | 0.03349 | 0.72344 | 0.03306 | 0.0718 |
| CIR | 2 | 0.69325 | 0.03555 | 0.67434 | 0.03232 | 0.3885 |
| CIR | 3 | 0.71343 | 0.03488 | 0.70046 | 0.0311 | 0.5585 |
| CIR | 4 | 0.73027 | 0.0343 | 0.69534 | 0.03107 | 0.11 |
| CIR | 5 | 0.70888 | 0.03736 | 0.6733 | 0.03395 | 0.1278 |
| CIR | 1 | 0.76397 | 0.03243 | 0.73497 | 0.03081 | 0.1079 |
| CIR | 2 | 0.78369 | 0.02951 | 0.73877 | 0.02951 | 0.0127 |
| CIR | 3 | 0.7925 | 0.02913 | 0.75917 | 0.02826 | 0.0873 |
| CIR | 4 | 0.79087 | 0.03005 | 0.76938 | 0.02819 | 0.2908 |
| CIR | 5 | 0.78821 | 0.03159 | 0.7656 | 0.02987 | 0.2977 |
| CIR | 1 | 0.76498 | 0.03447 | 0.7302 | 0.03313 | 0.0787 |
| CIR | 2 | 0.75739 | 0.0325 | 0.72415 | 0.03073 | 0.0821 |
| CIR | 3 | 0.75722 | 0.03232 | 0.71624 | 0.03062 | 0.038 |
| CIR | 4 | 0.75379 | 0.03318 | 0.70999 | 0.03129 | 0.0313 |
| CIR | 5 | 0.75741 | 0.03412 | 0.70399 | 0.03276 | 0.0109 |

## Table S3. Distribution of genomic aberrations included in the machine learning model, in the external validation cohort.

| **Genomic aberration / gene mutation** | **Number of patients** | **% of cohort** |
| --- | --- | --- |
| Normal karyotype | 395 | 55.32 |
| *NPM1* | 269 | 37.68 |
| *DNMT3A* | 204 | 28.57 |
| *FLT3*-ITD | 133 | 18.63 |
| *NRAS* | 121 | 16.95 |
| Complex karyotype | 93 | 13.03 |
| *IDH2* | 89 | 12.46 |
| *FLT3*-TKD | 84 | 11.76 |
| *TET2* | 82 | 11.48 |
| +8 | 67 | 9.38 |
| *PTPN11* | 66 | 9.24 |
| t(8;21) | 66 | 9.24 |
| *RUNX1* | 62 | 8.68 |
| inv(16)/t(16;16) | 58 | 8.12 |
| *IDH1* | 57 | 7.98 |
| *WT1* | 54 | 7.56 |
| *SRSF2* | 53 | 7.42 |
| *KIT* | 49 | 6.86 |
| Monosomal karyotype | 46 | 6.44 |
| *KRAS* | 45 | 6.3 |
| *STAG2* | 34 | 4.76 |
| *ASXL1* | 33 | 4.62 |
| bZIP in-frame mutated *CEBPA* | 30 | 4.2 |
| *BCOR* | 29 | 4.06 |
| Marker chromosome | 29 | 4.06 |
| *TP53* | 29 | 4.06 |
| Other *CEBPA* mutations | 28 | 3.92 |
| -Y | 27 | 3.78 |
| del(9q) | 22 | 3.08 |
| -5/del(5q) | 22 | 3.08 |
| -7 | 21 | 2.94 |
| t(v;11q23.3) | 19 | 2.66 |
| *U2AF1* | 18 | 2.52 |
| *EZH2* | 17 | 2.38 |
| +22 | 17 | 2.38 |
| -17/abn(17p) | 16 | 2.24 |
| del(7q) | 14 | 1.96 |
| +11 | 13 | 1.82 |
| -12/abn(12p) | 11 | 1.54 |
| -X | 11 | 1.54 |
| t(9;11) | 11 | 1.54 |
| -17 | 10 | 1.4 |
| +13 | 10 | 1.4 |
| +21 | 10 | 1.4 |
| -18 | 9 | 1.26 |
| +4 | 9 | 1.26 |
| *SF3B1* | 9 | 1.26 |
| *MECOM*r | 7 | 0.98 |
| *JAK2* | 7 | 0.98 |
| -12 | 6 | 0.84 |
| -20 | 6 | 0.84 |
| -21 | 6 | 0.84 |
| -5 | 6 | 0.84 |
| +10 | 6 | 0.84 |
| +6 | 6 | 0.84 |
| del(20q) | 5 | 0.7 |
| -13 | 5 | 0.7 |
| -19 | 5 | 0.7 |
| +19 | 5 | 0.7 |
| add(7q) | 4 | 0.56 |
| del(11q) | 4 | 0.56 |
| -16 | 4 | 0.56 |
| -22 | 4 | 0.56 |
| -3 | 4 | 0.56 |
| +1 | 4 | 0.56 |
| +15 | 4 | 0.56 |
| +9 | 4 | 0.56 |
| -8 | 3 | 0.42 |
| t(6;9) | 3 | 0.42 |
| del(13q) | 2 | 0.28 |
| *ZRSR2* | 2 | 0.28 |
| -11 | 1 | 0.14 |

## Table S4. Comparison of predictive performance, measured by the area under the receiver operating curve (AUC(t)) of HARMONY machine learning model, based on Bayesian Additive Regression Trees (BART), compared to European LeukemiaNet (ELN) 2022 risk classification in the external validation cohort.

| **Outcome type** | **Time (years)** | **AUC(t) (BART)** | **Standard error (BART)** | **AUC(t) (ELN2022)** | **Standard error (ELN2022)** | **p-value** |
| --- | --- | --- | --- | --- | --- | --- |
| CIR | 1 | 0.6986 | 0.0214 | 0.63572 | 0.0214 | <0.0001 |
| CIR | 2 | 0.69841 | 0.02047 | 0.65871 | 0.01978 | 0.0012 |
| CIR | 3 | 0.69965 | 0.02081 | 0.6618 | 0.01982 | 0.0021 |
| CIR | 4 | 0.71631 | 0.02116 | 0.66385 | 0.02037 | <0.0001 |
| CIR | 5 | 0.71813 | 0.02222 | 0.6722 | 0.02089 | 0.0004 |
| OS | 1 | 0.77721 | 0.02124 | 0.69857 | 0.02207 | <0.0001 |
| OS | 2 | 0.76304 | 0.01916 | 0.70373 | 0.01956 | <0.0001 |
| OS | 3 | 0.76407 | 0.01845 | 0.71727 | 0.01874 | 0.0001 |
| OS | 4 | 0.75792 | 0.01867 | 0.71133 | 0.01892 | 0.0001 |
| OS | 5 | 0.75531 | 0.01932 | 0.7136 | 0.01935 | 0.0007 |
| RFS | 1 | 0.69836 | 0.01995 | 0.64669 | 0.01956 | <0.0001 |
| RFS | 2 | 0.71958 | 0.01908 | 0.67687 | 0.0187 | 0.0001 |
| RFS | 3 | 0.71305 | 0.01946 | 0.67262 | 0.01891 | 0.0003 |
| RFS | 4 | 0.72045 | 0.01978 | 0.67322 | 0.01953 | <0.0001 |
| RFS | 5 | 0.71742 | 0.02088 | 0.67917 | 0.02015 | 0.0014 |

## Table S5. Comparison of predictive performance, measured by concordance index (C-index) of HARMONY machine learning model, based on Bayesian Additive Regression Trees (BART), compared to European LeukemiaNet (ELN) 2022 risk classification.

| **Outcome type** | **Cohort** | **C-index (BART)** | **Standard error (BART)** | **C-index (ELN2022)** | **Standard error (ELN2022)** |
| --- | --- | --- | --- | --- | --- |
| OS | Training (10-fold CV) | 0.699 | 0.022 | 0.654 | 0.021 |
| RFS | Training (10-fold CV) | 0.686 | 0.02 | 0.65 | 0.019 |
| CIR | Training (10-fold CV) | 0.661 | 0.021 | 0.633 | 0.022 |
| OS | External validation | 0.712 | 0.0138 | 0.668 | 0.0133 |
| RFS | External validation | 0.655 | 0.0128 | 0.618 | 0.0122 |
| CIR | External validation | 0.705 | 0.0115 | 0.670 | 0.0128 |
